# Seroprevalence of arboviruses in Ecuador: Implications for improved surveillance

**DOI:** 10.7705/biomedica.5623

**Published:** 2021-06-15

**Authors:** Ernesto Gutiérrez-Vera, Leandro Patiño, Martha Castillo-Segovia, Víctor Mora- Valencia, Julio Montesdeoca-Agurto, Mary Regato-Arrata

**Affiliations:** 1 Departamento de Virología, Instituto Nacional de Investigación en Salud Pública “Dr. Leopoldo Izquieta Pérez”, Guayaquil, Ecuador Departamento de Virología Instituto Nacional de Investigación en Salud Pública “Dr. Leopoldo Izquieta Pérez” Guayaquil Ecuador; 2 Dirección Técnica de Investigación, Desarrollo e Innovación, Instituto Nacional de Investigación en Salud Pública “Dr. Leopoldo Izquieta Pérez”, Guayaquil, Ecuador Dirección Técnica de Investigación, Desarrollo e Innovación Instituto Nacional de Investigación en Salud Pública “Dr. Leopoldo Izquieta Pérez” Guayaquil Ecuador; 3 Escuela de Medicina, Universidad de Especialidades Espíritu Santo, Samborondón, Ecuador Universidad de Especialidades Espíritu Santo Escuela de Medicina Universidad de Especialidades Espíritu Santo Samborondón Ecuador; 4 Laboratorio Clínico, Hospital General Guasmo Sur, Guayaquil, Ecuador Laboratorio Clínico Hospital General Guasmo Sur Guayaquil Ecuador; 5 Centro de Referencia Nacional de Virus Exantemáticos, Gastroentéricos y Transmitidos por vectores, Instituto Nacional de Investigación en Salud Pública “Dr. Leopoldo Izquieta Pérez”, Guayaquil, Ecuador Centro de Referencia Nacional de Virus Exantemáticos, Gastroentéricos y Transmitidos por vectores Instituto Nacional de Investigación en Salud Pública “Dr. Leopoldo Izquieta Pérez” Guayaquil Ecuador; ¥ Retired in 2012 Retired in 2012 Ecuador

**Keywords:** Arbovirus, yellow fever virus, West Nile virus, dengue virus, encephalitis virus, Venezuelan equine, encephalitis virus, Eastern equine., arbovirus, virus de la fiebre amarilla, virus del Nilo occidental, virus del dengue, virus de la encefalitis equina venezolana, virus de la encefalitis equina del este

## Abstract

**Introduction:**

Arthropod-borne viruses (arboviruses) cause morbidity and mortality in humans and domestic animals worldwide. The percentage of population immunity or susceptibility to these viruses in Ecuador is unknown.

**Objectives:**

To investigate the proportion of Ecuadorian populations with IgG antibodies (Abs) (past exposure/immunity) and IgM Abs (current exposure) against flaviviruses and alphaviruses and to study the activity of these viruses in Ecuador.

**Materials and methods:**

During 2009-2011, we conducted a serosurvey for selected arboviruses in humans (n=1,842), equines (n=149), and sentinel hamsters (n=84) at two coastal locations and one in the Amazon basin (Eastern Ecuador) using enzyme-linked immunosorbent assay and hemagglutination inhibition test.

**Results:**

From 20.63% to 63.61% of humans showed IgG-antibodies for the flaviviruses: Dengue virus (DENV), yellow fever virus (YFV) Saint Louis encephalitis virus, and West Nile virus (WNV); from 4.67% to 8.63% showed IgG-Abs for the alphaviruses: Venezuelan

equine encephalitis virus, eastern equine encephalitis virus, and western equine encephalitis virus. IgM-Abs were found for DENV and WNV. Equines and hamsters showed antibodies to alphaviruses in all locations; two hamsters seroconverted to YFV in the Amazonia.

**Conclusions:**

The results show a YFV vaccination history and suggest the activity of arboviruses not included in the current surveillance scheme. Enhanced arbovirus and mosquito surveillance, as well as continued YFV vaccination and evaluation of its coverage/ effectiveness, are recommended.

## Resumen

**Introducción.:**

Los virus transmitidos por artrópodos (arbovirus) causan morbilidad y mortalidad en humanos y animales domésticos mundialmente. Se desconoce el porcentaje de inmunidad o vulnerabilidad de la población ecuatoriana ante estos virus.

**Objetivos.:**

Investigar la proporción de poblaciones ecuatorianas con anticuerpos IgG (exposición o inmunidad pasada) y anticuerpos IgM (exposición reciente) contra flavivirus y alfavirus, e investigar su actividad en Ecuador.

**Materiales y métodos.:**

Entre 2009 y 2011, se llevó a cabo una encuesta serológica para arbovirus en humanos (n=1.842), equinos (n=149) y hámsters centinela (n=84) en dos localidades costeras y en una en la Amazonía, utilizando la prueba ELISA (*Enzyme-Linked ImmunoSorbent Assay*) y la prueba de inhibición de la hemaglutinación.

**Resultados.:**

Entre el 20,63 y el 63,61 % de los humanos registraron IgG contra el virus del dengue (DENV), el de la fiebre amarilla (YFV), el de la encefalitis de San Luis y el del Nilo Occidental (WNV); entre 4,67 y 8,63 % tenían IgG para los virus de la encefalitis equina venezolana, de la encefalitis equina del este y de la encefalitis equina del oeste.

Se encontró IgM para DENV y WNV. En los equinos y en los hámsters se encontraron anticuerpos contra alfavirus en todas las localidades muestreadas; dos hámsters mostraron seroconversión a YFV en la Amazonía.

**Conclusiones.:**

Los resultados del estudio evidenciaron los antecedentes de vacunación contra el YFV y sugieren la actividad de arbovirus no incluidos en el esquema de vigilancia actual. Se recomienda ampliar la vigilancia de arbovirus y mosquitos, continuar con la vacunación contra el YFV, y evaluar su cobertura y efectividad.

Arbovirus is an ecological term applied to viruses transmitted by arthropods, particularly mosquitoes and ticks ^1^. Most arboviruses are maintained in enzootic cycles and are transmitted between mosquitos and vertebrates. Humans become infected and may develop diseases ranging from subclinical or mild infections to systemic febrile illness, hemorrhagic fever, and meningoencephalitis ^1^. Examples of arboviral diseases include yellow fever, Zika, and dengue fever, which have reemerged or increased their geographic range causing public health emergencies and urging governments to enhance their surveillance programs and research ^2^. Around 534 arboviruses have been described, and at least 135 of them are of medical concern ^1^^,^^3^. Almost all arboviruses have RNA genomes and are classified into nine families and nine genera ^1^^,^^4^; arbovirus species of current medical importance in South America belong to *Flavivirus* (*Flaviviridae*), *Alphavirus* (*Togaviridae*), and *Orthobunyavirus* (*Perybunyaviridae*) genera ^4^.

In Ecuador, three flaviviruses *(*yellow fever virus, YFV, dengue virus, DENV, and Zika virus, ZIKV) and two alphaviruses *(*Venezuelan equine encephalitis virus, VEEV, and Chikungunya virus, CHIKV) have caused epidemic outbreaks ^5^^-^^9^. YFV has two well-recognized transmission cycles: urban and sylvatic ^10^. Recognized urban epidemics occurred in 1842, 1853-1856, 1867-1869, 1877-1878 and 1880 ^5^. Urban YF was eradicated in Ecuador in 1919 ^11^, but sylvatic YF has been documented since 1949 in Amazonian basin provinces ^12^^,^^13^. Vaccination campaigns against YFV have been conducted in Ecuador since 1944, particularly in the Amazonian and coastal provinces. DENV caused the first epidemic in 1988 ^6^ and currently, it is the most commonly detected arbovirus ^14^. ZIKV and CHIKV are more recent introductions; the first epidemics by these viruses occurred in 2014 and 2016, respectively. VEEV has caused two major epidemics, one in 1969 and the other in 1972; as usual for this virus, epidemic outbreaks were preceded by an epizootic event in equines ^7^^,^^15^. According to the Centers for Disease Control and Prevention (CDC), the 1969 outbreak caused 20,000 cases and 200 fatalities in Ecuador ^16^. Subsequently, a major outbreak of VEEV was reported in Guatemala, Central America, northern México, and Texas in the United States of America. The introduction of the epidemic subtype from Ecuador to Guatemala was speculated, but no convincing mechanism was identified ^17^. Later, it was shown that the different outbreaks originated from a residual live virus in incompletely formalin-inactivated vaccines ^18^^,^^19^.

Other arboviruses of medical concern have also been reported in Ecuador. They include the Saint Louis encephalitis virus (SLEV), the West Nile virus (WNV), and the Ilheus virus (ILHV) belonging to the *Flavivirus* genus; the eastern equine encephalitis virus (EEEV), the western equine encephalitis virus (WEEV), and the Mayaro virus (MAYV) from the *Alphavirus* genus, and the Oropouche virus (OROV) belonging to the *Orthobunyavirus* genus ^20^^-^^24^. SLEV, EEEV, and WEEV were isolated from sentinel hamsters and mosquitoes between 1974 and 1978 ^20^. Then, SLEV as well as ILHV, OROV, MAYV, and VEEV were reported after the detection of specific antibodies (Abs) in febrile humans sampled in the Ecuadorian Amazonia in 1997 and between 2001 and 2004 ^23^^-^^25^. ILHV and OROV were later isolated from febrile patients of the Ecuadorian Coast and the Amazonia, respectively ^22^^,^^24^. WNV and EEEV have been reported by the detection of specific Abs in horses of the Ecuadorean Coast: WNV from healthy equines sampled between 2007 and 2009 and EEEV from two equines showing neurological signs sampled in 2013 ^21^^,^^26^. Eight other enzootic arboviruses have been isolated in Ecuador and named according to the local geographic region where they were found, but little is known about their medical importance ^20^. One example is the “Playas virus”, of which two strains (75V3066 and 75V938) are classified as Cache Valley virus and one (75V5758) as Maguari virus, both belonging to the genus *Orthobunyavirus* and associated with human disease ^27^.

Serosurveys are a key tool for determining the proportion of individuals exposed and of those with immunity to infectious agents, as well as for the detection of subclinical infections ^28^. In Ecuador, little is known about these aspects of arboviruses. The surveillance activities of the *Ministerio de Salud Pública* - MSP (Ministry of Public Health) are focused on the diagnosis of DENV, YFV, CHIKV, and ZIKV, mainly using nucleic acid detection; other arboviruses are surveyed using generic primers, a method with intrinsic limitations that may lead to missing arboviruses of medical concern. In this study, we aimed to investigate the proportion of three Ecuadorian populations with IgG Abs (past exposure/immunity) against selected flaviviruses and alphaviruses. This population included conscripts of a military fort of the Ecuadorian Amazonia recently vaccinated against YFV with the “YFV-17D” vaccine. We also determined the percentage of IgM Abs (recent exposure) against DENV and WNV in febrile and healthy individuals to look for clinical and subclinical infections. Finally, we emphasize the importance of widening and strengthening arbovirus surveillance in Ecuador.

## Materials and methods

### Ethical statement

This project was approved by the institutional review board of the *Instituto Nacional de Higiene y Medicina Tropical “Dr. Leopoldo Izquieta Pérez”* (INHMTLI) now *Instituto Nacional de Investigación en Salud Pública*. Human samples were collected under verbal informed consent. Domestic animals were sampled given owner consent and supervision. Hamsters were used as sentinel species in the field and suckling mice “Balb/C4” were required for inoculation of hamster tissue and mosquito pools. Hamsters and mice were grown and housed at the laboratory animal facility of INHMTLI, which has adequate infrastructure to guarantee animal welfare and dedicated personnel for cage cleaning and animal feeding. After exposure/inoculation, the animals were euthanized using chloroform as an inhaled anesthetic. All these procedures were performed in strict accordance with the institutional guidelines for the care and use of laboratory animals. We followed the ethical guidelines of the Declaration of Helsinki and the “Public Health Service Policy on Humane Care and Use of Laboratory Animals” published by the National Institute of Health of the United States of America.

### Sampling and sampling location

**Figure 1:**
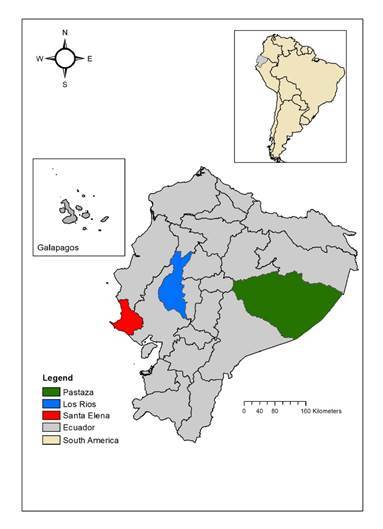
Sampling sites and number of human samples collected. Santa Elena Province-Manglaralto (N=496), Los Ríos Province-Vinces (N=474), and Pastaza Province-Puyo (N=872: 293 civilians and 579 conscripts)

This study comprised serum samples of humans and equines for arbovirus seroprevalence analysis, serum samples of hamsters for arbovirus seroconversion analysis, mosquito pools, and hamster tissues for viral isolation. Sampling was performed from 2009 to 2011 at three locations in Ecuador, two in the coastal region (Santa Elena Province-Manglaralto and Los Ríos Province-Vinces) and one in the Amazonia basin (Pastaza Province- Puyo) (figure 1). Manglaralto and Vinces were chosen for the presence of marshes and wetlands that contribute to the presence of mosquitoes. Puyo is in the Amazon rainforest, where many arboviruses circulate in their natural cycles. Hamsters were exposed as sentinel species at each sampling location for one week. During the study period, each location was visited six times, more or less every three months. The number of human samples collected by location is shown in figure 1. Detailed information on the number of human samples, sentinel hamsters, equines, and mosquitoes collected by location and date is shown in table S1.

The procedure for sampling humans and domestic animals is described by Beaty, *et al*. ^29^. The human population sampled was expected to have been vaccinated against YFV in different immunization campaigns. In one location (Puyo), we sampled conscripts of the Jungle Brigade 17 Pastaza (Amazonas Military Fort), who had all recently been vaccinated with YFV- 17D. Two different groups of conscripts were sampled three times during the year of their stay in the military fort to evaluate IgG Abs against YFV and their seroprevalence to other flaviviruses.

Mosquitoes were trapped with CDC light traps supplemented with CO_2_^30^ and then frozen in dry ice and transported to the laboratory.

### Laboratory analysis

Seroprevalence/seroconversion to arbovirus was evaluated mainly through the analysis of IgG Abs. For humans, each sample was analyzed against a battery of arboviruses comprising DENV, YFV, SLEV, WNV, EEEV, WEEV, and VEEV. Only monotypic results were considered positive for the corresponding virus. IgG Abs against DENV and WNV were evaluated by commercial ELISA methods of PANBIO (Windsor, Queensland, Australia, specificity 81.6 - 97.2% and sensitivity 73.9 - 85.1%) and FOCUS (Cypress, CA, USA, specificity 97% and sensitivity 36%), respectively. IgG Abs against YFV, SLEV, EEEV, WEEV, and VEEV were evaluated with in-house ELISAs following Johnson, *et al.*^31^. Sucrose-acetone-extracted suckling mouse brain antigens and positive serum against these viruses were donated by the CDC’s National Center of Infectious Diseases, Division of Vector-Borne Infectious Diseases (Fort Collins, CO, United States of America). IgM Abs against WNV and DENV were also analyzed in individuals who were febrile at the time of sampling or reported to be febrile one or two weeks before sampling (table S2). Since arboviruses also cause subclinical infections, IgM Abs against these viruses were further analyzed in a subset of nonfebrile individuals using commercial ELISAs of PANBIO (specificity: 85.4 - 98.9%; sensitivity: 46.6 - 64.7%) and FOCUS (specificity: 100%; sensitivity: 100%), as described above. Positive and negative serum samples were included for the respective ELISA test. The IBM SPSS Statistics package for Windows, version XX (IBM Corp., Armonk, N.Y., USA), was used for frequency analysis of monotypic samples according to individual age.

Equine and hamster IgG Abs were evaluated with the hemagglutination inhibition (HI) test on kaolin-adsorbed sera according to the technique of Clarke, *et al.*^32^, adapted to microtiter plates and following the procedures described in the document *Técnicas de laboratorio para el diagnóstico y la caracterización de los virus del dengue*^33^. The test was conducted for EEEV, WEEV, and VEEV, and nine hamsters were also evaluated for YFV. We used the same antigens donated by the CDC. A shortage of antigens did not allow testing other arboviruses or more hamsters for YFV. An HI titer of 1:20 or higher inhibiting hemagglutination produced by four antigen units was considered positive. Hamsters ^5^^-^^7^ (table S1) were analyzed before and after their exposure at the sampling site ^34^. After exposure, hamsters were euthanized and their tissues (brain, heart, lungs, kidneys, liver, spleen, and skeletal muscle) were processed for IC inoculation in suckling mice Balb/C4 and analyzed by RT-PCR.

Mosquitoes were identified, classified, and grouped by species. Spearman correlation was carried out using SPSS to test the relationship between the number of mosquitoes captured by species and location and the proportion of human samples showing IgG Abs reactive against the arboviruses tested. This nonparametric test was chosen given that the proportion of samples having Abs was calculated on categorical data (yes/no). Mosquitoes pooled by species in groups of 50 were crushed in a sterile environment and processed for intracerebral (IC) inoculation in suckling mice Balb/C4 and RT-PCR analysis.

RT-PCR for flaviviruses and alphaviruses was conducted on samples from febrile humans, hamster tissues, and mosquito pools according to Ayers, *et al.*^35^ and Sánchez-Seco, *et al.*^36^, respectively.

## Results

**Figure S1:**
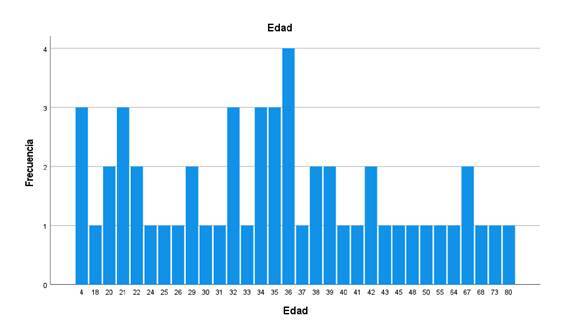
Frequency of monotypic samples of WNV according to patient age

**Figure S2:**
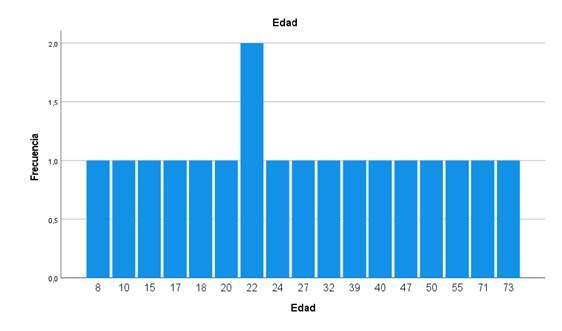
Frequency of monotypic samples of SLEV according to patient age

**Figure S3:**
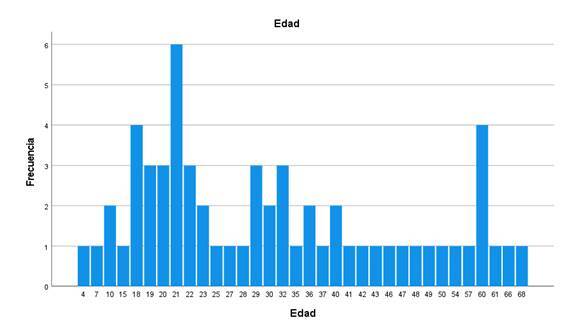
Frequency of monotypic samples of VEEV according to patient age

**Figure S4:**
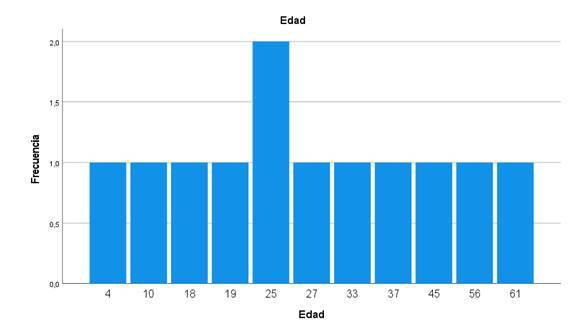
Frequency of monotypic samples of WEEV according to according to patient age.

**Figure S5:**
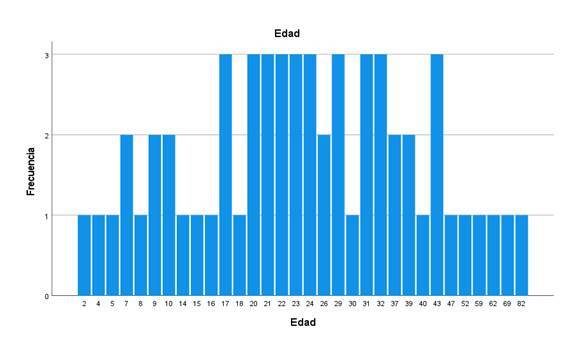
Frequency of monotypic samples of EEEV according to according to patient age

Analysis of IgG Abs against selected arboviruses was performed on 1,842 human sera. We found Abs for all the viruses investigated: DENV, YFV, SLEV, WNV, EEEV, WEEV, and VEEV. The percentages of Abs against different arboviruses in 1,263 samples (without including conscripts) are shown in Table 1. The flaviviruses showed the highest seroprevalence with WNV (63.61%), followed by YFV (62.67%), SLEV (52.29%), and DENV (20.63%); the alphaviruses showed the lowest prevalence, particularly WEEV (4.67%). Some samples reacted with only one of the viruses tested (monotypic samples): WNV: 58, SLEV: 20, EEEV: 54, VEEV: 64, and WEEV: 18. The frequency of monotypic samples according to the age of the patient is shown in figures S1-S5. Individuals between 2 and 82 years of age showed monotypic reactions.

**Table 1:** Percentages of Abs against the arbovirus evaluated in the civilian population (conscripts not included), N=1,223 to 1,263. The number of samples analyzed varied depending on sample or reagent availability.

Virus	Percentage	(Positivies/analyzed)
DENV	20,63	(260/1,260)
YFV	62,67	(769/1,227)
SLEV	51,29	(635/1,238)*
WNV	63,61	(778/1,223)*
EEEV	8,63	(109/1,263)*
WEEV	4,67	(59/1,263)*
VEEV	7,60	(96/1,263)*

DENV: Dengue virus, YFV: Yellow fever Virus, SLEV: Saint Louis encephalitis Virus, WNV: West Nile encephalitis Virus, EEEV: Eastern equine encephalitis virus, WEEV: Western equine encephalitis virus, VEEV: Venezuelan equine encephalitis virus

* There were monotipic results as follows: WNV 69 (34 Vinces, 21 Manglaralto, and 14 Puyo); SLEV 20 (5 Vinces, 2 Manglaralto, 13 Puyo); EEEV (11 Vinces, 39 Manglaralto, 4 Puyo); WEEV (3 Vinces, 10 Manglaralto, 5 Puyo); VEEV (25 Vinces, 23 Manglaralto, 16 Puyo).

**Figure 2:**
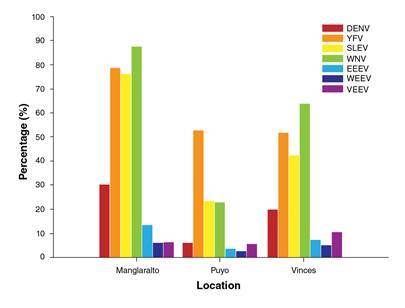
Percentage of civilian people with IgG Abs against selected flaviviruses and alphaviruses in three locations of Ecuador, method used: ELISA. DENV: Dengue virus, YFV: Yellow fever virus, SLEV: Saint Louis encephalitis virus, WNV: West Nile virus, EEEV: Eastern equine encephalitis virus, WEEV: Western equine encephalitis virus, VEEV: Venezuelan equine encephalitis virus

The results of IgG analysis by location are shown in figure 2. A total of 474 samples were studied in Vinces; the highest seroprevalence was found for WNV, 63.59% (276/434), and the lowest was for WEEV, 4.85% (23/474). In Manglaralto, we screened 496 samples. The highest seroprevalence was found for WNV, 87.7% (435/496), and the lowest for WEEV, 5.84% (29/496). In Puyo, we analyzed 872 samples comprising 293 samples from the civilian population and 579 from conscripts. For the civilian population, the highest seroprevalence was found for YFV, 52.75% (153/290), and the lowest for WEEV, 2.38% (7/293). Puyo is one of the Amazonian provinces subject to regular YFV vaccination.

**Figure 3:**
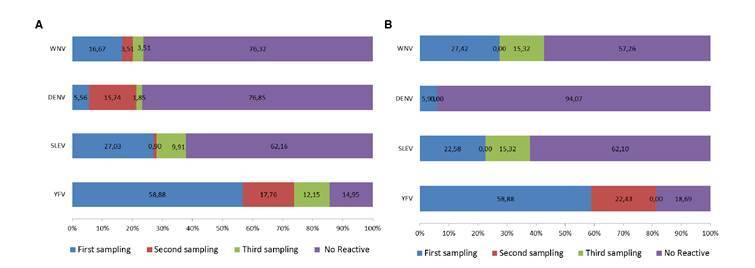
Percentage of conscripts developing IgG Abs against YFV and other selected flaviviruses during the year after being vaccinated with YFV- 17D. Two groups of conscripts (a and b) from the Amazonian Military Fort “Selva Pastaza 17” were sampled and analyzed for the mentioned virus at three different times of the year. The percentage of conscripts with Abs against YFV increased with time. Method used, ELISA. WNV: West Nile virus; DENV: Dengue virus, SLEV: Saint Louis encephalitis virus, YFV: Yellow fever virus. a) First group of conscripts. b) Second group of conscripts. Each bar shows the increasing percentage of Abs for each virus during the three sampling periods.

The percentage of conscripts with IgG Abs against arbovirus is shown in figure 3a and b. Two groups (group 1: 61 individuals and group 2: 67 individuals) recently vaccinated against YFV were sampled three times. For group 1, the percentage of response to the vaccine was 85.05% and between 23.15% and 37.84% showed Abs against DENV, WNV, and SLEV. In group 2, 81.31% showed Abs against YFV, and between 5.90% and 42.74% showed Abs against DENV, WNV, and SLEV.

A total of 102 individuals were registered as febrile and analyzed for IgM Abs against WNV and DENV, and the detailed results are shown in Table S2. Four individuals from Puyo (30.76%-4/13) showed IgM Abs reactive against DENV, one in the civilian population, and three in the second group of conscripts (two were positive in the second sampling and the other in the third sampling). Two samples showed monotypic IgM Abs-reactive against WNV: one from a 19-year-old female in Vinces (1.66%-1/60) and another from a 53- year old woman in Manglaralto (5.55% -1/18).

A total of 1,343 samples of nonfebrile individuals (including conscripts) were analyzed for IgM Abs against DENV of which 5.06% (18/356) in Vinces, 2.42% (12/496) in Manglaralto, and 7.94% (39/491) in Puyo showed Abs. A total of 893 samples of nonfebrile individuals (including conscripts) analyzed for IgM Abs against WNV demonstrated 0.20% (1/496) positivity from Manglaralto and 0.20% (1/491) positivity from Puyo.

The HI results are shown in tables S3 and S4; 149 equines were analyzed. Hemagglutination-inhibiting Abs were found for all the antigens evaluated at serum dilutions of 1:20-1:1280. Counting the monotypic results and the highest dilution showing complete inhibition of hemagglutination in heterotypic reactions, 1.34% (2/149) of equines demonstrated Abs to EEEV, 5.37% (8/149) to WEEV, and 6.04% (9/149) to VEEV across all sampling sites. Abs to EEEV were found in Vinces, Abs to WEEV were detected in Vinces and Manglaralto, and Abs against VEEV were detected in all the locations sampled. Hemagglutination-inhibiting Abs at higher serum dilutions were found for WEEV in one animal in Vinces (1:1280) (table S3).

A total of 84 hamsters were used as sentinels. After exposure in the field, 22.62% of them demonstrated Abs to EEEV (18.51% -5/27 in Vinces, 15.63% -5/32 in Manglaralto, and 25.71% -9/35 in Puyo), 20.24% showed Abs to VEEV (7.41% -2/27 in Vinces, 31.25% -10/32 in Manglaralto, and 20% -7/35 in Puyo), and 2.38% seroconverted to YFV (22.22% -2/9 in Puyo) (table S4). Hemagglutination-inhibiting Abs at higher serum dilutions were found for YFV in one hamster in Puyo (1:640) and for EEEV in two hamsters in Manglaralto (1:1280). None of the hamsters became sick.

A total of 73,505 mosquitoes were captured. The number by locality and species is described in table S5. The most common species was *Culex (Mel)* spp*.* (N=63.057)*.* The most abundant by location were *Culex (Mel)* spp*.* in Vinces (N=62.736)*, Aedes (Stg) aegypti* in Manglaralto (N=912), and *Coquillettidia venezuelensis* in Puyo (N=160)*.* Correlation results are shown in table S6*.* There was a very strong negative correlation (ρ=-1, p≥0.1) between *Psorophora ferox* and samples with Abs against DENV, SLEV, WNV, EEV, WEEV, and between *C. venezuelensis* or *A. albimanus* and samples showing Abs for YFV (ρ=-1 p≥0.1). A strong positive correlation was found between *Mansonia pseudotitilians/indubitans* or *Anopheles (Nys) triannulatus* and the proportion of individuals showing Abs against VEEV (ρ=1, p≥0.1). There was a positive correlation between *A. aegypti* and DENV, SLEV, WNV, EEV, and WEEV, but it was not significant. A list of mosquito species identified in this study and the arboviruses they harbor according to viral isolation studies elsewhere is shown in table S7.

RT-PCR analysis of 128 mosquito pools and 119 hamster tissues did not lead to the amplification of *Flavivirus* or *Alphavirus* RNA.

We inoculated 807 suckling Balb/C mice, 576 with hamster tissue and 231 with suspensions of polled mosquitoes. No virus was isolated.

## Discussion

Serosurvey of arboviruses is constrained by Ab cross-reactivity because of antigenic similarities between members of the same virus family cocirculating in the same region. The plaque reduction neutralization test (PRNT) is the gold standard for the specific detection of Abs, but it is difficult to implement in resource-limited countries. ELISA and HIA tests are valuable alternatives when monotypic results are accounted for. Using these techniques, we report the percentage of individuals with IgG Abs reactive against DENV, YFV, WNV, SLEV, EEEV, WEEV, and VEEV and with IgM Abs reactive against DENV and WNV. We found monotypic results for WNV, SLEV, and each of the encephalitis viruses evaluated while the frequency was found in individuals of different ages. We also demonstrated the seroconversion of naive sentinel hamsters to the EEEV and VEEV in all sampling locations and for YFV in Amazonia. Mosquito genera recognized as vectors of arboviruses were found in all locations studied. We discuss our findings in the context of arboviruses whose activity has been confirmed in Ecuador and with the current surveillance schemes.

Monotypic IgG Abs against WNV were found in Manglaralto-Santa Elena Province and Vinces-Los Ríos Province (N=58: 34 Vinces, 24 Manglaralto). IgM Abs against this virus were also found in two IgG-positive samples, one from Manglaralto and one from Vinces. The result in Vinces is consistent with the finding of specific IgG Abs against this virus in equines sampled during 2007-2009 by Coello ^21^; thus, it is probable that WNV infection occurred in humans in this region during these years. The confirmation of human infection would require the use of PNRT; however, these findings warrant further investigation of the activity of this virus in humans, vectors, and potential reservoirs. Serosurvey of birds in Guayaquil (a coastal city around 100 km from Vinces) did not find evidence of WNV infection at this location during 2011 ^37^. Analysis of SLEV showed 20 monotypic results (2 in Manglaralto, 13 in Puyo, and 5 in Vinces). From 1974 to 1978, SLEV was isolated on the Ecuadorian Coast from *Culex nigripalpus* mosquitoes (isolated 76 V-1177 and 24684) collected in Huertas Negras-Tenguel/Guayas and Puerto Nuevo- Vinces/Los Ríos and sentinel hamsters (isolated 78V-5682) exposed in Playas/Guayas (20). From 2001 to 2004, specific IgM Abs against this virus were found in febrile patients sampled from Ecuadorian Amazonia ^25^.

A higher percentage of Abs was found against YFV, which was probably due to YFV vaccination history. Overall, the civilian population showed ~60% Abs against YFV, with Puyo showing a higher percentage of civilian people with YFV Abs (52.75%). In conscripts, the percentage of Abs was between 81.31% and 85.05%, which is not optimal but agrees with vaccine efficacy percentages ^38^^,^^39^. Two hamsters seroconverted to YFV in Puyo, which added to the cases of sylvatic yellow fever occurring in Ecuadorian Amazonia showing the active circulation of the virus in this region and the presence of susceptible people. According to information provided by the MSP, mass preventive YFV vaccination in Ecuador was performed in 1944, 1975, 1997, 2005, and 2016, and data about routine immunization of infants have been publicly available since 2007 ^40^. A worldwide analysis of YFV vaccination from 1970 to 2016 ^41^ shows a cumulative vaccination coverage of ~ 60% in the Ecuadorian Amazonia and of ~ 30% in the entire country and recommends reaching the 80% threshold to prevent or control outbreaks.

Regarding *Alphavirus,* we found IgG Abs reactive against VEEV, EEEV, and WEEV in humans and equines and Abs to VEEV and EEEV in naive hamsters. All these viruses were isolated in Ecuador by Calisher, *et al.* between 1974 and 1978 ^20^. VEEV comprises six antigenic subtypes (I-VI) of which I-AB and I-C are associated with epizootic/epidemic activity in equines and humans while other serotypes circulate in natural enzootic cycles ^42^. The epidemic subtype IB (now I-AB) and the enzootic 1-D have been reported in Ecuador; IB, in particular, was reported from a horse showing neurological disease in 1944 and during the outbreak of 1969 ^7^. The isolates from 1969 were antigenically similar to those causing outbreaks in Central and North America ^42^ and likely originated from incompletely formalin-inactivated vaccines ^18^^,^^19^. IgM Abs against VEEV have been detected in Ecuadorian individuals since 1958, then in 1960, and from febrile patients sampled during 2000-2004 ^7^. The emergence of epizootic/epidemic strains from enzootic VEEV has also been shown and justifies surveillance attention ^43^^,^^44^. For EEEV, specific IgM Abs were detected in two equines showing neurological signs from Chongón, Guayas Province, in 2013 ^26^. EEEV circulating in South America is now known as *Madariaga virus*^45^ and has been linked to neurologic disease in humans and horses ^46^. WEEV has not been reported to cause epizootics or epidemics in Ecuador.

As regards mosquitoes, most of the species/genera identified in this study are known vectors of arboviruses elsewhere (table S7). For example, some *Culex* species are vectors of WNV, SLEV, and VEEV, and the presence of these mosquitoes in all locations is consistent with the finding of Abs against the viruses they transmit. *A. aegypti,* the main vector of DENV, was found only in Manglaralto, although Abs against dengue were found in all locations. The absence of this mosquito species in the other locations studied could be due to sampling bias; in Manglaralto, traps were placed near human settlements and in Puyo and Vinces they were placed in more forested areas. Correlation data between species and the proportion of human individuals showing IgG Abs against arbovirus should be interpreted with caution because few locations were analyzed (N=3). *Anopheles nuneztovari* has not been reported in Ecuador before, but this species is difficult to identify and will need further confirmation. Anopheles are not usual vectors for arboviruses, but the finding of unreported species highlights the importance of carrying out more studies on mosquitoes.

By 2009-2012, arboviral surveillance in Ecuador comprised DENV and YFV through ELISA and nucleic acid detection. After the arrival of ZIKV and CHIKV and the confirmation of OROV, the surveillance was extended to these viruses. Following our results, a percentage of negative samples are currently analyzed for VEEV, EEEV, and MAYV by RT-PCR. Given the emergence and reemergence of arboviral diseases worldwide and their continuous spread, we strongly recommend widening the analysis and surveillance of other arboviruses, especially those isolated previously or detected in febrile patients in the country. These must include the arbovirus surveyed here but also the ILHV and the Playas virus. This last virus, isolated in Ecuador in 1974-1978, is genetically similar to the Cache Valley virus and the Maguari virus, which have been reported to cause human disease. Likewise, we emphasize the need for updated studies and surveillance of mosquito ecology and their vectorial capacity for arbovirus transmission.

We also urge for the establishment of a set of recommendations given by the Global Arbovirus Group of Experts (GAGE) ^47^, which include strengthening laboratory capacity through the implementation of multiplex diagnostic tests reliable for differential diagnosis, access to viral isolation, sequencing, and genotyping, and encouraging the development of research programs to study the mechanism of arboviral evolution, emergence, and dispersal. Implementation of viral isolation would allow phenotypic characterization and availability of reagents for serological studies, which, in its turn, will reduce the dependence on international reference centers for local diagnosis ^48^. A constraint of this research was the lack of a local method for the specific analysis of Abs, such as PNRT, which requires cell culture, a procedure used intermittently by the MSP surveillance laboratories. Sequencing and genotyping could now be approached through genomic technologies, whole genome sequencing, and the simultaneous analysis of several samples making it useful for rapid outbreak investigations, viral phylogeography, and evolutionary studies ^49^^-^^51^. Improving epidemiological surveillance and implementation of the recommendations provided by the GAGE could be achieved locally and regionally by promoting collaboration between public laboratories and academia, as recommended by Miranda, *et al.*^52^; this approach would be particularly useful for developing countries where infrastructure and technical and economic resources are scarce.

## SUPPLEMENTARY FILES

**Table S1:** Number of samples of humans, sentinel hamsters, equines, and mosquitoes collected by location and date

Location*	Collection date	Samples			
		Humans	Sentinel Hamsters	Esquines	Mosquitoes
Vinces	9-13/3/09	115	5	0	278
Manglaralto	25-27/3/09	54	5	15	266
Vinces	25-30/5/09	120	5	20	722
Puyo	7-11/9/09	244	7	0	34
Manglaralto	21-25/9/09	129	7	21	243
Puyo	26-30/10/09	149	7	10	8
Vinces	1-5/02/2010	39	6	0	65,318
Manglaralto	22-26/03/2010	46	5	17	281
Puyo	3-7/05/2010	94	5	0	84
Vinces	19-23/07/2010	57	5	11	1,301
Manglaralto	20-24/09/2010	105	6	14	308
Puyo	18-22/10/2010	177	6	5	30
Puyo	14-18/2/2011	103	5	2	15
Vinces	21-25/3/2011	43	5	9	3,212
Manglaralto	16-20/5/2011	77	5	13	82
Puyo	22-26/8/2011	105	6	0	65
Manglaralto	26-30/9/2011	85	5	14	709
Vinces	21-25/11/2011	100	5	37	549
Total		1,842	100	186	73,505

* The samples were collected from the following sites: Vinces: Hospital Nicolás Cotto Infante, Subcentro de Salud San Lorenzo de Vinces, Isla de Bejucal, Subcentro de Salud Antonio Sotomayor, Abras de Mantequilla, El Recuerdo, Puerto Nuevo, Campo Alegre, Playones La Luz, Palenque, San Miguel de Palenque, Haciendas La María y El Rocío. Manglaralto: Hospital de Manglaralto, -Dos Mangas-, Santa María del Fiat, Haciendas San Francisco, El Edén y La Carmela, Fincas Lauricel Rodríguez, Manuel Baque y Benito Chiquito, - Olón-, Finca El Retiro, -La entrada-, Finca La Española: Puyo: Subcentro de Salud El Dorado, Fuerte Militar Amazonas-Brigada de Selva 17 Pastaza-Shell, Hospital Provincial de Puyo, Quesería 10 de Agosto, Recinto Fátima, Reserva de monos, Depósito de agua potable

**Table S2:** Number of febrile patients (n) per sampling location analyzed for IgM Abs against WNV and DENV and percentage of reactive samples

Location	n (female/ male)	WNV IgM* % of positives (number of positives/n)	DENV IgM % of positives (number of positives/n)
Vinces	69 (36/33)	1.66 (1/60)	0 (0/60)
Manglaralto	20 (16/4)	5.55 (1/18)	0 (0/18)
Puyo	13 (3/10)	0 (0/13)	30.76 (4/13)
Total	102 (55/47)	1.96 (2/102)	4.65 (4/86)

**Table S3:** Equines showing Abs to *Alphavirus* by HI test of N=149. EEEV: Eastern equine encephalitis virus; WEEV: Western equine encephalitis virus; VEEV: Venezuelan equine encephalitis virus. The reciprocal of the highest dilution of serum showing complete inhibition of hemagglutination is shown for each virus. *= monotypic reactions, ** highest dilution showing complete inhibition of hemagglutination in heterotypic reactions

Sample Code	Date	EEEV	WEEV	VEEV	Location
EGV789	24/09/2009	40	0	40	Manglaralto
EGV791	24/09/2009	40	0	40	Manglaralto
EGV 2239	28/09/2011	40	0	40	Manglaralto
EGV 2240	28/09/2011	20	0	20	Manglaralto
EGV 2241	28/09/2011	20	20	40**	Manglaralto
EGV1979	18/05/2011	0	20*	0	Manglaralto
EGV2010	19/05/2011	0	20*	0	Manglaralto
EGV2012	19/05/2011	0	20*	0	Manglaralto
EGV229	26/03/2009	0	0	20*	Manglaralto
EGV1145	25/03/2010	0	0	40*	Manglaralto
EGV 2262	29/09/2011	0	0	40*	Manglaralto
EGV321	27/05/2009	40	80	80	Vinces
EGV322	27/05/2009	40*	0	0	Vinces
EGV323	27/05/2009	80	80	80	Vinces
EGV324	27/05/2009	40	80	80	Vinces
EGV328	27/05/2009	160	80	160	Vinces
EGV329	27/05/2009	160	1,280**	80	Vinces
EGV330	27/05/2009	40	80	80	Vinces
EGV331	27/05/2009	160	320**	40	Vinces
EGV332	27/05/2009	40	320**	40	Vinces
EGV333	27/05/2009	40	320**	0	Vinces
EGV334	27/05/2009	40	160**	40	Vinces
EGV1292	20/07/2010	40*	0	0	Vinces
EGV318	27/05/2009	0	40	40	Vinces
EGV326	27/05/2009	0	20	20	Vinces
EGV335	27/05/2009	0	80	160**	Vinces
EGV1290	20/07/2010	0	0	2 0*	Vinces
EGV1885	23/03/2011	0	0	20*	Vinces
EGV1888	23/03/2011	0	0	40*	Vinces
EGV1711	21/10/2010	0	0	40*	Puyo

**Table S4:** Hamsters showing Abs against Alphavirus by HI test of N=84 after exposure in the field. EEEV: Eastern equine encephalitis virus; WEEV: Western equine encephalitis virus; VEEV: Venezuelan equine encephalitis virus. The reciprocal of the highest dilution of serum showing complete inhibition of hemagglutination is shown for each virus. *= monotypic reactions, ** highest dilution showing complete inhibition of hemagglutination in heterotypic reactions

Sample code	Date	EEEV	WEEV	VEEV	Location
EGV1163	25/03/2010	20*	0	0	Manglaralto
EGV1165	25/03/2010	20*	0	0	Manglaralto
EGV1513	24/09/2010	20	0	20	Manglaralto
EGV1514	24/09/2010	80*	0	0	Manglaralto
EGV1515	24/09/2010	80*	0	0	Manglaralto
EGV1516	24/09/2010	40**	0	20	Manglaralto
EGV1517	24/09/2010	20	0	20	Manglaralto
EGV1518	24/09/2010	40*	0	0	Manglaralto
EGV2018	31/05/2011	40	40	20	Manglaralto
EGV 2271	30/09/2011	20	0	40**	Manglaralto
EGV2019	31/05/2011	0	20	40**	Manglaralto
EGV829	25/09/2009	0	0	20*	Manglaralto
EGV830	25/09/2009	0	0	20*	Manglaralto
EGV832	25/09/2009	0	0	20*	Manglaralto
EGV1164	25/03/2010	0	0	20*	Manglaralto
EGV2015	31/05/2011	0	0	20*	Manglaralto
EGV2016	31/05/2011	0	0	40*	Manglaralto
EGV2017	31/05/2011	0	0	20*	Manglaralto
EGV 2273	30/09/2011	0	0	20*	Manglaralto
EGV1075	05/02/2010	20*	0	0	Vinces
EGV1077	05/02/2010	20*	0	0	Vinces
EGV1373	23/07/2010	20*	0	0	Vinces
EGV1374	23/07/2010	20	0	20	Vinces
EGV1376	23/07/2010	40*	0	0	Vinces
EGV1918	25/03/2011	20*	0	0	Vinces
EGV1078	05/02/2010	0	0	40*	Vinces
EGV1374	23/07/2010	20	0	20	Vinces
EGV1377	23/07/2010	0	0	20*	Vinces
EGV657	11/09/2009	1,280**	0	20	Puyo
EGV1280	07/05/2010	20*	0	0	Puyo
EGV1283	07/05/2010	40*	0	0	Puyo
EGV1284	07/05/2010	40*	0	0	Puyo
EGV1717	22/10/2010	40**	0	20	Puyo
EGV1718	22/10/2010	80**	0	20	Puyo
EGV1719	22/10/2010	40*	0	0	Puyo
EGV1720	22/10/2010	20*	0	0	Puyo
EGV 2149	01/09/2011	20*	0	0	Puyo
EGV653	11/09/2009	0	0	20*	Puyo
EGV655	11/09/2009	0	0	40*	Puyo
EGV658	11/09/2009	0	0	20*	Puyo
EGV1722	22/10/2010	0	0	20*	Puyo
EGV1744	16/02/2011	0	0	80*	Puyo
EGV1745	16/02/2011	0	0	40*	Puyo
EGV1837	17/02/2011	0	0	20*	Puyo

**Table S5:** Number of mosquitoes per genus and species captured in the locations studied

Species	Vinces	Manglaralto	Puyo	Total
*Trichoprosopon digitatum*	0	0	7	7
*Culex (Mel)* spp*.*	62,732	293	32	63,057
*Ochlerotatus serratus*	2,325	507	27	2,859
*Aedeomyia squamipennis*	261	1	0	262
*Psorophora (Jan) ferox*	1	0	5	6
*Mansonia pseudotitilians/indubitans*	4,234	72	0	4,306
*Coquillettidia venezuelensis*	1,476	0	160	1,636
*Aedes (Stg) aegypti*	0	912	0	912
*Anopheles (Nys) albimanus*	32	1	4	37
*Anopheles (Nys) triannulatus*	319	14	1	334
*Anopheles (Nys) pseudopunctipennis*	0	85	0	85
*Anopheles (Nys) nuneztovari*	0	4	0	4
Total	71,380	1,889	236	73,505

**Table S6:** Spearman correlation of the number of mosquitoes captured by species and the proportion of human samples showing IgG Abs reactive against DENV, SLEV, WNV, YFV, EEV, WEEV, and VEEV

Virus and statistics estimates					Mosquitoes especies											
					Td	C_sp	O_s	A_s	Ps_sp	M_sp	Cq_v	Ae_a	An_a	An_t	An_pse	An_nun
Spearman’s rho	DENV	Correlation coefficient			-0.866	0.500	0.500	0.500	-1,000**	0.500	-0.500	0.866	-0.500	0.500	0.866	0.866
		Sig. (2-tailed)			0.333	0.667	0.667	0.667		0.667	0.667	0.333	0.667	0.667	0.333	0.333
		N			3	3	3	3	3	3	3	3	3	3	3	3
		Sampling simulation	Bias Standard error		0.000	0.000	0.000	0.000	0.000	0.000	0.000	0.000	0.000	0.000	0.000	0.000
					0.000	0.000	0.000	0.000	0.000	0.000	0.000	0.000	0.000	0.000	0.000	0.000
			Confidence interval 95%	Lower limit	-0.866	0.500	0.500	0.500	-1.000	0.500	-0.500	0.866	-0.500	0.500	0.866	0.866
				Upper limit	-0.866	0.500	0.500	0.500	-1.000	0.500	-0.500	0.866	-0.500	0.500	0.866	0.866
	SLEV	Correlation coefficient			-0.866	0.500	0.500	0.500	-1,000**	0.500	-0.500	0.866	-0.500	0.500	0.866	0.866
		Sig. (2-tailed)			0.333	0.667	0.667	0.667		0.667	0.667	0.333	0.667	0.667	0.333	0.333
		N			3	3	3	3	3	3	3	3	3	3	3	3
		Sampling simulation	Bias Standard error		0.000	0.000	0.000	0.000	0.000	0.000	0.000	0.000	0.000	0.000	0.000	0.000
					0.000	0.000	0.000	0.000	0.000	0.000	0.000	0.000	0.000	0.000	0.000	0.000
			Confidence interval 95%	Lower limit	-0.866	0.500	0.500	0.500	-1.000	0.500	-0.500	0.866	-0.500	0.500	0.866	0.866
				Upper limit	-0.866	0.500	0.500	0.500	-1.000	0.500	-0.500	0.866	-0.500	0.500	0.866	0.866
																
	WNV	Correlation coefficient			-0.866	0.500	0.500	0.500	-1,000**	0.500	-0.500	0.866	-0.500	0.500	0.866	0.866
		Sig. (2-tailed)			0.333	0.667	0.667	0.667		0.667	0.667	0.333	0.667	0.667	0.333	0.333
		N			3	3	3	3	3	3	3	3	3	3	3	3
		Sampling simulation	Bias Standard error		0.000	0.000	0.000	0.000	0.000	0.000	0.000	0.000	0.000	0.000	0.000	0.000
					0.000	0.000	0.000	0.000	0.000	0.000	0.000	0.000	0.000	0.000	0.000	0.000
			Confidence interval 95%	Lower limit	-0.866	0.500	0.500	0.500	-1.000	0.500	-0.500	0.866	-0.500	0.500	0.866	0.866
				Upper limit	-0.866	0.500	0.500	0.500	-1.000	0.500	-0.500	0.866	-0.500	0.500	0.866	0.866
	YFV	Correlation coefficient			0.000	-0.500	-0.500	-0.500	-0.500	-0.500	-1,000**	0.866	-1,000**	-0.500	0.866	0.866
		Sig. (2-tailed)			1.000	0.667	0.667	0.667	0.667	0.667		0.333		0.667	0.333	0.333
		N			3	3	3	3	3	3	3	3	3	3	3	3
		Sampling simulation	Bias Standard error		0.000	0.000	0.000	0.000	0.000	0.000	0.000	0.000	0.000	0.000	0.000	0.000
					0.000	0.000	0.000	0.000	0.000	0.000	0.000	0.000	0.000	0.000	0.000	0.000
			Confidence interval 95%	Lower limit	0.000	-0.500	-0.500	-0.500	-0.500	-0.500	-1.000	0.866	-1.000	-0.500	0.866	0.866
				Upper limit	0.000	-0.500	-0.500	-0.500	-0.500	-0.500	-1.000	0.866	-1.000	-0.500	0.866	0.866
	EEEV	Correlation coefficient			-0.866	0.500	0.500	0.500	-1,000**	0.500	-0.500	0.866	-0.500	0.500	0.866	0.866
		Sig. (2-tailed)			0.333	0.667	0.667	0.667		0.667	0.667	0.333	0.667	0.667	0.333	0.333
		N			3	3	3	3	3	3	3	3	3	3	3	3
		Sampling simulation	Bias Standard error		0.000	0.000	0.000	0.000	0.000	0.000	0.000	0.000	0.000	0.000	0.000	0.000
					0.000	0.000	0.000	0.000	0.000	0.000	0.000	0.000	0.000	0.000	0.000	0.000
			Confidence interval 95%	Lower limit	-0.866	0.500	0.500	0.500	-1.000	0.500	-0.500	0.866	-0.500	0.500	0.866	0.866
				Upper limit	-0.866	0.500	0.500	0.500	-1.000	0.500	-0.500	0.866	-0.500	0.500	0.866	0.866
	WEEV	Correlation coefficient			-0.866	0.500	0.500	0.500	-1,000**	0.500	-0.500	0.866	-0.500	0.500	0.866	0.866
		Sig. (2-tailed)			0.333	0.667	0.667	0.667		0.667	0.667	0.333	0.667	0.667	0.333	0.333
		N			3	3	3	3	3	3	3	3	3	3	3	3
		Sampling simulation	Bias Standard error		0.000	0.000	0.000	0.000	0.000	0.000	0.000	0.000	0.000	0.000	0.000	0.000
					0.000	0.000	0.000	0.000	0.000	0.000	0.000	0.000	0.000	0.000	0.000	0.000
			Confidence interval 95%	Lower limit	-0.866	0.500	0.500	0.500	-1.000	0.500	-0.500	0.866	-0.500	0.500	0.866	0.866
				Upper limit	-0.866	0.500	0.500	0.500	-1.000	0.500	-0.500	0.866	-0.500	0.500	0.866	0.866
	VEEV	Correlation coefficient			-0.866	1,000**	1,000**	1,000**	-0.500	1,000**	0.500	0.000	0.500	1,000**	0.000	0.000
		Sig. (2-tailed)			0.333				0.667		0.667	1.000	0.667		1.000	1.000
		N			3	3	3	3	3	3	3	3	3	3	3	3
		Sampling simulation	Bias Standard error		0.000	0.000	0.000	0.000	0.000	0.000	0.000	0.000	0.000	0.000	0.000	0.000
					0.000	0.000	0.000	0.000	0.000	0.000	0.000	0.000	0.000	0.000	0.000	0.000
			Confidence interval 95%	Lower limit	-0.866	1.000	1.000	1.000	-0.500	1.000	0.500	0.000	0.500	1.000	0.000	0.000
			Upper limit	-0.866	1.000	1.000	1.000	-0.500	1.000	0.500	0.000	0.500	1.000	0.000	0.000

** Correlation is significant at 0,01 Sig. (2-tailed).

Simulation was based on 1,000 replicates.

**Table S7:** Mosquito species identified in this study and the arboviruses they harbor according to viral isolation studies elsewhere*

Mosquitos species	Arbovirus carried	Reference
*Trichoprosopon digitatum*	Pixuna virus and Wyeomyia virus. Bussuquara, SLEV and ILHV have been isolated from mixed pools that included this species.	(1-2)
*Culex (Mel) spp.*	WNV, VEEV, VESL, Japanese Encephalitis virus	(3)
*Ochlerotatus serratus*	OROV, YFV, ILHV	(4-6)
*Aedeomyia squamipennis*	Gamboa virus, VEEV	(7,8)
*Psorophora (Jan) ferox*	Rocio virus	9
*Mansonia pseudotitilians/indubitans*	VEEV	10
*Coquillettidia venezuelensis*	MAYV, OROV y SLEV, WNV	(11-12)
*Aedes (Stg) aegypti*	DENV, YFV, ZKV, CHKV	13
*Anopheles (Nys) albimanus*	Not known	
*Anopheles (Nys) triannulatus*	Not known	
*Anopheles (Nys) pseudopunctipennis*	Not known	
*Anopheles (Nys) nuneztovari*	Not known	
TOTAL	Not known	

*This is not an exhaustive list of arboviruses carried by mosquitoes
