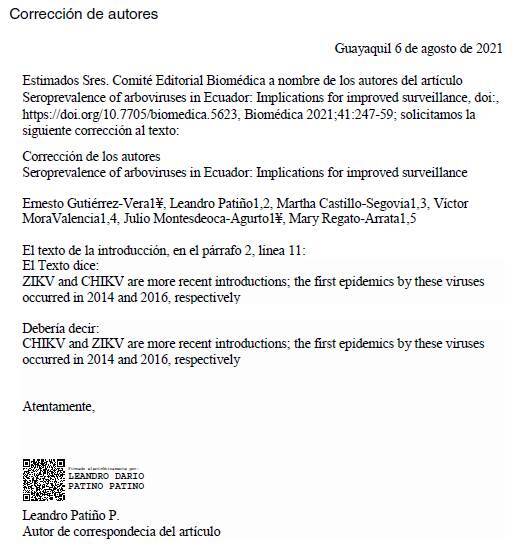# Corrección de autores

**Published:** 2021-09-22

**Figure:**